# The impact of narrative nursing intervention on nursing outcomes of patients undergoing laparoscopic uterine fibroid surgery: A retrospective study

**DOI:** 10.1097/MD.0000000000044505

**Published:** 2025-09-12

**Authors:** Xiaofeng Long, Cai Tang, Yi Zhou, Huanyu Liang, Liqi Zhang, Liqin Zhu

**Affiliations:** a Legislative Affairs Office, Hunan Provincial People’s Hospital (The First Affiliated Hospital of Hunan Normal University), Changsha, China; b Department of Gynecology, Hunan Provincial People’s Hospital (The First Affiliated Hospital of Hunan Normal University), Changsha, China.

**Keywords:** anxiety, depression, narrative nursing, quality of life, uterine fibroid surgery

## Abstract

This study aims to analyze the impact of narrative nursing intervention methods on patients undergoing laparoscopic uterine fibroid surgery. A retrospective analysis was conducted on 234 patients with uterine fibroids who underwent laparoscopic surgery in the gynecology department from January 2023 to December 2024. Patients were divided into a narrative nursing group and a control group based on whether they received a narrative nursing intervention. The nursing effects of the 2 groups of patients were compared and analyzed, including negative emotions, nursing satisfaction, and postoperative recovery. There were 114 patients in control group and 120 in narrative nursing intervention group. The self-rated anxiety scale, self-rated depression scale, Hamilton anxiety scale, and Hamilton depression scale of patients in the narrative nursing group after nursing were significantly lower than those in the control group (*P* < .05). Patient satisfaction in the narrative nursing group was significantly higher than in the control nursing group (*P* < .05). The average hospitalization time and hospitalization costs of patients in the narrative nursing group were significantly lower than those in the control nursing group (*P* < .05). The narrative nursing group had higher nursing satisfaction (*P* < .05). Narrative nursing intervention has good application value in patients undergoing laparoscopic uterine fibroid surgery. Patients who adopt narrative nursing intervention have better postoperative recovery effects, can alleviate postoperative anxiety and depression, and improve patient nursing satisfaction.

## 1. Introduction

Uterine fibroids are the most common benign tumors in females. Common symptoms of uterine fibroids include pain, bleeding, emotional distress, urinary problems, and sexual dysfunction.^[1,2]^ When the medication cannot alleviate symptoms, surgical treatment is recommended. Surgery, as the main treatment method, can also bring about varying degrees of physiological and psychological burden on patients, affecting postoperative recovery.^[3]^ Laparoscopic surgery is a common minimally invasive surgical form in clinical practice and has a relatively wide range of applications in the treatment of gynecological diseases. For patients with uterine fibroids, myomectomy, and hysterectomy can be chosen based on the location and size of the fibroids.^[4]^ Although laparoscopic surgery has less trauma and faster recovery compared to traditional open surgery, psychological pressure during surgery and discomfort during rehabilitation can also have a direct impact on the overall prognosis and recovery of patients.^[5]^ Therefore, the focus of clinical research is how to effectively alleviate patients’ psychological pressure, provide patient satisfaction with nursing services, and promote rapid postoperative recovery of patients.^[6]^

With the development of nursing concepts, more and more research has begun to focus on the effects beyond traditional physical therapy, such as the impact of psychological and social factors on the rehabilitation of patients.^[7]^ Narrative nursing intervention is a scientific nursing method based on the concept of humanistic nursing. Its core idea is to emphasize the effective interaction of “knowledge-emotion-intention” in clinical nursing, providing support for nursing staff to understand patients’ conditions and enrich clinical intervention pathways. Narrative nursing intervention, as an emerging humanistic nursing model, mainly guides patients to describe their condition, experiences, and feelings, helps them vent their emotions, reduces their psychological pressure, and improves self-efficacy through cognitive restructuring, thus promoting physical and mental health.^[8–11]^ There are some excellent application effects in various diseases, but the impact of narrative nursing on laparoscopic uterine fibroids patients is currently unclear. This study aims to retrospectively analyze the effect of narrative nursing implementation in patients with uterine fibroids treated with laparoscopic surgery in the gynecology department of Hunan Provincial People’s Hospital (The First Affiliated Hospital of Hunan Normal University) from January 2023 to December 2024, and to evaluate the impact of narrative nursing intervention methods on patients undergoing laparoscopic uterine fibroids surgery.

## 2. Materials and methods

### 2.1. Clinical data

Retrospective analysis of clinical data from patients with uterine fibroids admitted to the gynecology department of Hunan Provincial People’s Hospital (The First Affiliated Hospital of Hunan Normal University) from January 2023 to December 2024. Inclusion criteria: age over 18 years old; diagnosed as a patient with uterine fibroids through clinical symptoms, ultrasound, and imaging examinations; no previous mental illness; perform laparoscopic surgery for treatment. Exclusion criteria: accompanied by severe heart, liver, and kidney disease; malignant tumor patients; individuals with severe mental illness or cognitive impairment; prior to the start of the study, narrative caregivers had been received. This study was approved by the Ethics Committee of Hunan Provincial People’s Hospital (The First Affiliated Hospital of Hunan Normal University) (No: 2025-113). The informed consent forms were obtained from all enrolled patients.

### 2.2. Intervention methods

All patients underwent laparoscopic surgery. The surgery method included single port laparoscope and multi-port laparoscope. The surgical types included myomectomy and hysterectomy.

The control group implemented routine nursing interventions, including management of perioperative complications, medication management, etc. Patients received guidance on the daily diet and exercise management of the patients during the perioperative period, including introducing precautions for the daily diet during the perioperative period, such as eating light foods, increasing the intake of fresh fruits and vegetables appropriately, and avoiding constipation. The patient’s vital signs after surgery were monitored regularly and the possible complications were detected and treated promptly. The patients received appropriate pain relief measures based on the patient’s pain score. After surgery, patients received guidance on respiratory exercises to promote recovery from abdominal function.

The narrative nursing group measures include the following specific content. The first step was to establish a trusting relationship between nurses and patients. Before nursing started, nurses established a deep trust relationship with patients. Nurses could demonstrate the sincerity of nursing services through proper communication to make patients feel connected and understood. The nurses could then gradually explore more personal and sensitive topics through regular communication to allow patients to express themselves in a safe and stress-free environment. For example, nursing staff could first share their personal experiences, reduce the defensive mentality of patients, and encourage patients to open up. The second step was narrative guidance. Finding the appropriate narrative method was the key to improving nursing effectiveness. And cultural differences between patients would directly affect the implementation measures of narrative nursing. Therefore, nurses could use narrative methods that correspond to the personality and communication style of patients with uterine fibroids based on the evaluation of their physical condition. In accordance with patient privacy, nurses could present their case information in a storytelling way. And nurses could raise appropriate questions such as “How long do you think the postoperative recovery period is?” or “Do you think emotions can be controlled after surgery?.” By exchanging and analyzing the case data, both the nurse and the patient could discuss the key points of the story. By posing carefully designed questions, nurses could guide patients to narrate their own stories; it inspires patients’ memories and emotions. For example, ``Is there a special moment during your treatment that makes you feel hopeful and energized?’’ Based on motivational questions, nurses could help patients recall and share emotional experiences and encourage them to express themselves in a comfortable way, verbally, in writing, or through artistic works. The third step was to implement narrative deconstruction. When the nurse and patient communicated about the plot of the story, the nursing staff listened to the patient’s description of the story and provided positive psychological support, guiding them to quickly break out of traditional thinking patterns and helping them reshape their optimistic mindset. When patients showed pessimism and lack of confidence in the story plot, nursing staff could guide them to accelerate the deconstruction of the story plot, break the original ending of the story, and guide their behavior and thinking patterns with positive and constructive results. After the patients shared their stories, the nursing staff could guide deep reflection and discussion, focusing on how the patients understand and handle related experiences. Nursing staff helped patients explore the meaning behind the story and uncover clues to personal growth by asking questions such as “Do you feel any different when recalling this experience?” or “Has this experience changed your perception of health or life?” During the narrative nursing period, nursing staff demonstrated sufficient empathy and understanding for patients, strengthened communication and exchanged with patients through empathetic means. It was particularly pointed out that narrative nursing required attention to the methods and techniques of explanation. Nursing staff used vivid language to guide patients in describing stories and encouraged them to describe their emotions and feelings in detail, such as “describe the environment around you at that moment, what did you feel.” Nursing staff could help patients describe problems more vividly, specifically. During the description process, feedback and confirmation were used to strengthen the patient’s narrative, such as restating the patient’s expression to express his understanding of the patient. Nursing staff could also introduce creative expression, such as painting or writing narratives, to help patients present content that cannot be expressed in words, while also better understanding the patient’s inner thoughts. The final step was to record and share. After obtaining the patient’s consent, nurses could record the patient’s story and share it with other patients. This not only allowed patients to gain a sense of recognition, but also had a positive impact on other patients based on their individual stories, leveraging the mutual help of the patient group. All nurses who implement narrative nursing have completed narrative nursing training at our medical institution and obtained qualification certificates. Each patient in the narrative nursing group completed narrative nursing at admission, before surgery, after surgery, and before discharge. Each communication lasted about 30 to 60 minutes depending on the patient’s condition. The detailed typical implementation case could be found in File S1, Supplemental Digital Content, https://links.lww.com/MD/P952.

### 2.3. Observation indicators and judgment criteria

Negative emotions, quality of life (QOL), nursing satisfaction and rehabilitation status between 2 groups of patients were compared.

Negative emotions were assessed using the self-rated anxiety scale (SAS), self-rated depression scale (SDS), Hamilton anxiety scale and Hamilton depression scale (HAMD). SAS contained 20 items that reflect subjective feelings of anxiety, each of which was graded into 4 levels based on the frequency of symptom appearance, with 15 positive questions and 5 negative questions. The cutoff value for the SAS standard score was 50 points; 50 to 59 points indicated mild anxiety, 60 to 69 moderate, and ≥69 severe anxiety.^[12]^ The SDS contained 20 elements that reflect subjective feelings of depression, each of which was graded into 4 levels based on the frequency of symptom occurrence, with 10 positive scores and 10 negative scores. The cutoff value for the SDS standard score was 53 points; 53 to 62 points indicated mild depression, 63 to 72 moderate, and ≥72 severe anxiety.^[13]^ Hamilton anxiety scale (HAMA) classified anxiety factors into 2 main types: somatic factors and psychogenic factors. A total score of 29 may indicate severe anxiety, 21 points indicate obvious anxiety, 14 points indicate anxiety. Exceeding 7 points may indicate anxiety and if it is <7 points, there were no symptoms of anxiety. HAMD consisted of 24 items, with a total score of <35 points, which may indicate severe depression. Exceeding 20 points may indicate mild or moderate depression. If it is <8 points, the patient did not have depressive symptoms.^[14]^ QOL assessment was carried out using the 36-item short-form (SF-36) scale. The SF-36 scale included 36 items that evaluated 8 dimensions of QOL related to health.^[15]^ Nursing satisfaction was based on the self-made nursing satisfaction survey. The self-made nursing satisfaction survey was rated from 0 to 100 points. A score >80 points indicated very satisfied, 60 to 80 points indicated satisfied, and <60 points indicated dissatisfied.

### 2.4. Statistical methods

Statistical analysis was performed using Statistical Product and Service Solutions (SPSS) software (version 25.0, SPSS Inc., Chicago). Measurement data were expressed as mean ± standard deviation (*x* ± s), and intergroup comparisons were conducted using *t* test or repeated measures analysis of variance; count data was presented in terms of the number of cases (%) and the comparison between groups is conducted using the Chi square test. *P* < .05 indicates a statistically significant difference.

## 3. Results

### 3.1. Basic information of 2 sets of clinical data

**Figure 1.**
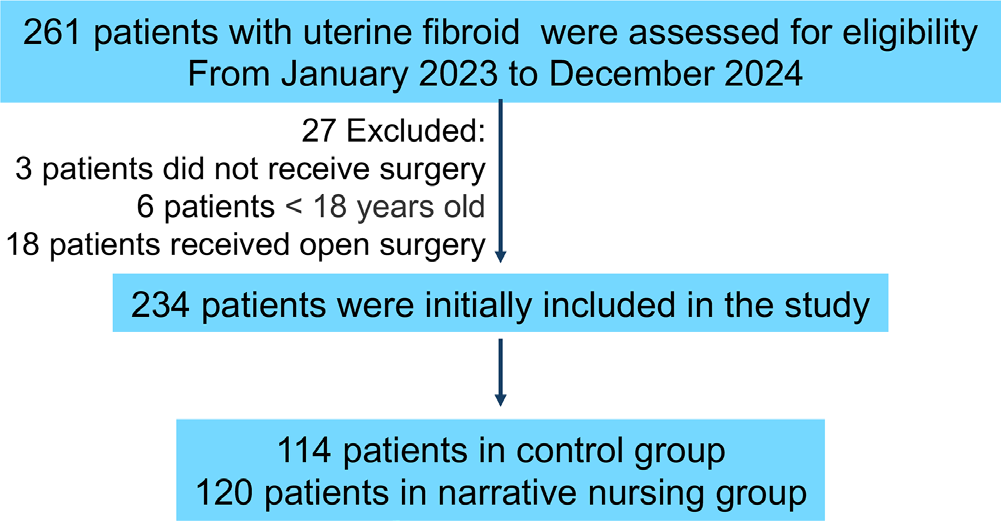
Flow chart of patients’ enrollment.

Between January 2023 and December 2024, 261 patients with uterine fibroids were evaluated for eligibility. Finally, 234 patients were included in this study, with 114 patients in the control group and 120 patients in the narrative nursing group (Fig. 1). The baseline of 2 groups of patients including age, marital status, education level, monthly income, employment, residence, etc were presented in Table 1. There were 78 patients underwent myomectomy and 34 patients underwent hysterectomy in the control group. While 76 patients underwent myomectomy and 44 patients underwent hysterectomy in the narrative nursing group (*P* = .190). Moreover, all the other basic information had no difference between the 2 groups. From this table, it was also shown that uterine fibroids were more common in married women in the 40s, and were not closely related to education level, monthly income, or residence in rural or urban areas. Most of the diseases previously combined were hypertension and diabetes, and most of the operations were cesarean sections. The uterine fibroids were mainly multiple intramural tumors larger than 5 cm, located mainly in the anterior and lateral walls of the uterus. And the proportion of single port laparoscopy was gradually increasing, accounting for more than a third of the patients.

**Table 1:** The baseline of 2 groups of patients undergoing laparoscopic uterine fibroid surgery.

Parameters	Control group (N = 114)	Narrative nursing group (N = 120)	*t*/χ^2^	*P*-value
Age (years)	45.06 ± 6.75	46.38 ± 7.80	0.812	.368
*Marital status*				
Unmarried	5 (4.39%)	6 (5.00%)	0.302	.860
Married	106 (92.98%)	112 (93.33%)		
Divorced	3 (2.63%)	2 (1.67%)		
*Education level*				
Junior high school or lower	49 (42.98%)	51 (42.50%)	1.598	.450
Senior high school	25 (21.93%)	34 (28.33%)		
College or higher	40 (35.09%)	35 (29.17%)		
*Monthly income (yuan*)				
<3000	68 (59.65%)	76 (63.33%)	0.297	.593
≥3000	46 (40.35%)	44 (36.67%)		
*Employment*				
No	31 (27.19%)	41 (34.17%)	1.338	.260
Yes	83 (72.81%)	79 (65.83%)		
*Residence*				
Rural areas	56 (49.12%)	53 (44.17%)	0.799	.432
Urban areas	58 (50.88%)	67 (55.83%)		
*Payment method*				
Self-funded	3 (2.63%)	2 (1.67%)	0.261	.677
Medical insurance	111 (97.37%)	118 (98.33%)		
*Comorbidities*				
No	91 (79.82%)	93 (77.50%)	0.188	.750
Yes	23 (20.18%)	27 (22.50%)		
*Previous surgeries history*				
No	56 (49.12%)	55 (45.83%)	0.399	.600
Yes	58 (50.88%)	65 (54.17%)		
*Diameter of fibroids (cm*)				
<5	32 (28.07%)	35 (29.17%)	0.106	.775
≥5	82 (71.93%)	85 (70.83%)		
*Number of fibroids*				
Single	36 (31.58%)	47 (39.17%)	1.474	.274
Multiple	78 (68.42%)	73 (60.83%)		
*Types of fibroids*				
Submucosal	12 (10.53%)	14 (11.66%)	1.653	.412
Intramural myoma	67 (58.77%)	77 (64.17%)		
Subserosa membrane	16 (14.06%)	11 (9.17%)		
Mixed type	19 (16.66%)	18 (15.00%)		
*Location of fibroids*				
Anterior wall	43 (37.72%)	53 (44.17%)	1.969	.742
Rear wall	38 (33.33%)	39 (32.50%)		
Side wall	1 (0.88%)	2 (1.67%)		
Bottom	6 (5.26%)	4 (3.33%)		
Mixed type	26 (22.81%)	22 (18.33%)		
*Surgery method*				
LESS	40 (35.09%)	38 (31.67%)	0.308	.583
MPLS	74 (64.91%)	82 (68.33%)		
*Surgical type*				
Myomectomy	78 (69.64%)	76 (63.33%)	1.033	.190
Hysterectomy	34 (30.36%)	44 (36.67%)		

LESS = single port laparoscope, MPLS = multi-port laparoscope.

### 3.2. Narrative nursing had a better effect of improving negative emotions

**Figure 2.**
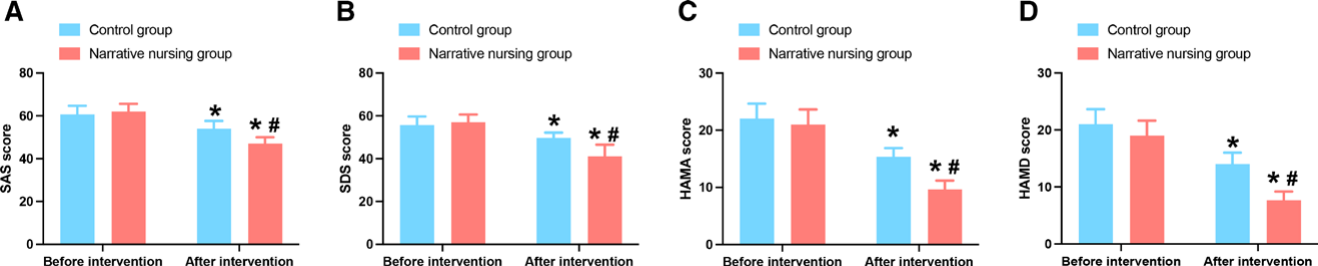
Negative emotions of 2 groups of patients undergoing laparoscopic uterine fibroid surgery. (A) SAS scores of patients. (B) SDS scores of patients. (C) HAMA scores of patients. (D) HAMD scores of patients. Note: * presents *P* < .05 compared with before intervention. # presents *P* < .05 compared with the control group. HAMA = Hamilton anxiety scale, HAMD = Hamilton depression scale, SAS = self-rated anxiety scale, SDS = self-rated depression scale.

Negative emotions were evaluated using SAS score, SDS score, HAMA score, and HAMD score. The results of the SAS scale showed that preoperative patients had mild to moderate anxiety and some even reached severe anxiety. However, after the intervention, the anxiety of the patients was relieved (*P* < .05). And compared to the control group, the anxiety score of the narrative nursing group was lower (*P* < .05) (Fig. 2A). The results of the SDS scale showed that the preoperative patients had mild depression. However, after the intervention, the depression of the patients was relieved (*P* < .05). And compared to the control group, the depression score of the narrative nursing group was lower (*P* < .05) (Fig. 2B). The results of the HAMA scale were consistent with the results of the SAS scale (Fig. 2C). The results of the HAMD scale were consistent with the results of the SDS scale (Fig. 2D). All results indicated that narrative nursing had a better improvement effect on negative emotions.

### 3.3. Narrative nursing had a better effect of improvement on postoperative rehabilitation

**Figure 3.**
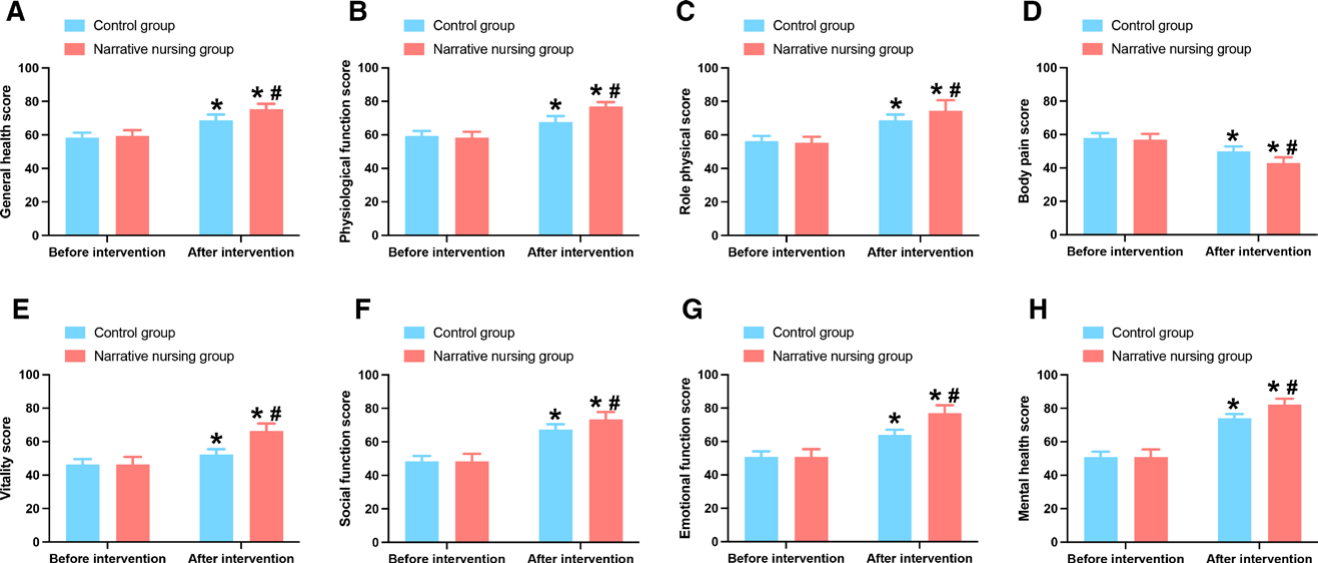
Quality of life of 2 groups of patients undergoing laparoscopic uterine fibroid surgery. (A) Overall health scores of patients. (B) Physical function scores of patients. (C) Role-physical scores of patients. (D) Bodily pain scores of patients. (E) Vitality scores of patients. (F) Social function scores of patients. (G) Role-emotional scores of patients. (H) Mental health scores of patients. Note: * presents *P* < .05 compared with before intervention. # presents *P* < .05 compared with the control group.

The operative time of 2 groups was 128.25 ± 47.95 minutes for the control group and 119.91 ± 47.99 minutes for the narrative nursing group, respectively (*P* = .179). The indwelling time of the urinary catheter and the anal exhaust time were shorter in the narrative nursing group than in the control group (*P* < .05). Due to the better recovery of the narrative nursing group, their hospitalization days were shorter and hospitalization costs were lower (*P* < .05) (Table 2). SF-36 was used to evaluate the quality-of-life score of patients after discharge. The data showed that all patients improved compared to before treatment (*P* < .05) (Fig. 3). The narrative nursing group had higher QOL scores in all 8 dimensions (*P* < .05) (Fig. 3). All results showed that narrative nursing had a better improvement effect on postoperative rehabilitation.

**Table 2:** Analysis of effect of narrative nursing on patients undergoing laparoscopic uterine fibroid surgery.

Parameters	Control group	Narrative nursing group	*t*	*P*-value
Operative time (min)	128.25 ± 47.95	119.91 ± 47.99	2.784	.179
Indwelling time of urinary catheter (h)	30.30 ± 21.11	22.41 ± 19.14	8.045	.036
Anal exhaust time (h)	42.73 ± 21.00	35.20 ± 21.78	7.658	.039
Hospitalization days (d)	7.45 ± 2.42	6.37 ± 2.49	11.673	.028
Hospitalization expenses (yuan)	23,482 ± 5816.67	20,866 ± 5141.58	4.975	.043

### 3.4. The narrative nursing group had higher nursing satisfaction

**Figure 4.**
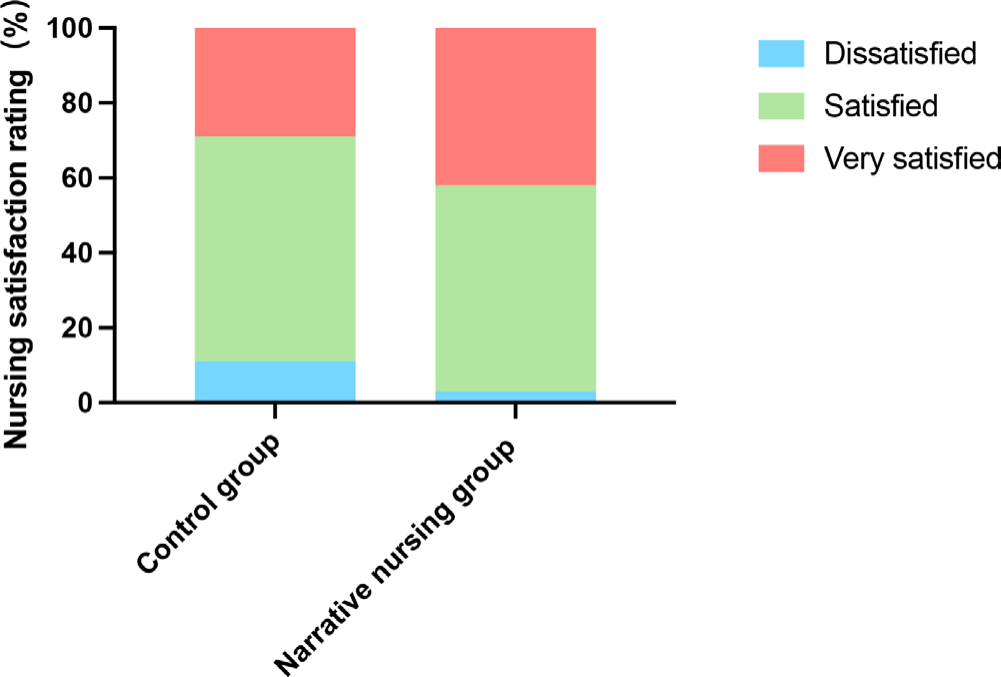
Nursing satisfaction rating of 2 groups of patients undergoing laparoscopic uterine fibroid surgery.

Nursing satisfaction was based on the self-made nursing satisfaction survey. There were 12 (10.53%) dissatisfied, 68 (59.65%) satisfied, and 34 (29.82%) very satisfied patients in the control group. Meanwhile, there were 3 (2.50%) patients unhappy, 66 (55.00%) satisfied, and 51 (42.50%) very satisfied patients in the narrative nursing group. The results showed that the narrative nursing group had greater nursing satisfaction (*P* < .05) (Fig. 4).

## 4. Discussion

Uterine fibroids are relatively common in clinical practice and can cause increased menstrual bleeding and anemia in patients, seriously affecting their physical health. Larger uterine fibroids can also cause stress symptoms, leading to bladder dysfunction such as urgency, frequency, and incontinence.^[16,17]^ Traditional treatment of uterine fibroids mainly involves open surgery. However, the risk of complications during hysterectomy is approximately 10%, and there is a risk of local recurrence and surgical-related complications during hysterectomy. In the past 30 years, with the advancement of instruments and technology, laparoscopic myomectomy has been developed. Compared to traditional open surgery, laparoscopic myomectomy has advantages such as shorter operation time, less trauma, and faster recovery.^[18,19]^ However, nursing care during and after surgery plays an important role and value in ensuring surgical effectiveness, promoting postoperative recovery, and reducing complications. Nursing services during laparoscopic surgery involve not only physiological monitoring and pain management prior to surgery, but also postoperative sign monitoring and prevention and control of complication.^[20,21]^ Overall, although traditional nursing services can meet the needs for surgical treatment, it is difficult to regulate patients’ negative emotions and their promotion of rehabilitation efficiency is limited. Therefore, it is very important to improve and optimize nursing models.

Narrative nursing intervention is a nursing method designed based on the relevant theories of evidence-based medicine, which belongs to a nursing measure with narrative as the basic concept. Narrative nursing intervention is a scientific method implemented through storytelling, communication, and other methods based on witnessing, understanding, experiencing and responding to patient pain. The focus is on promoting patient mental health and physical recovery through the narration and reflection of their experiences. The core of narrative nursing lies in understanding the unique life stories and disease experiences of each patient, releasing emotions through sharing stories, improving self-identity, and discovering the inner strength to cope with diseases. An increasing number of studies have confirmed the effectiveness of narrative nursing. For postoperative patients, bone or joint replacement patients can benefited from narrative nursing interventions because they increased nurse compliance and satisfaction, significantly improved QOL, decreased complication rates, and decreased pain levels.^[7]^ For acute diseases, acute pancreatitis patients significantly alleviated anxiety and ameliorated negative emotions after receiving narrative nursing programs.^[9]^ For family members of patients, narrative nursing can effectively alleviate negative emotions, reduce perceived stress, and improve nursing abilities of family members of children with biliary atresia.^[8]^ For chronic diseases, narrative nursing can greatly reduce negative emotions, alleviate anxiety, and improve confidence in treatment and QOL for malignant tumor patients undergoing chemotherapy.^[22]^ It can be seen that narrative nursing has a wide range of applications, mainly used to alleviate patients’ negative emotions and improve their QOL. This study also confirms the application value of narrative nursing in improving negative emotions in patients after uterine fibroids surgery. This broadens the application of narrative nursing and provides optional nursing strategies for patients with uterine fibroids.

Nursing staff can also deepen their own cognitive state and handle the patient’s disease narrative content, enabling effective reflection between both parties. The entire communication process can be filled with the experience of nursing staff of the disease, which can eliminate the patient’s negative emotions in a shorter period of time. In addition, narrative nursing can promote mutual support among patients. By sharing stories and listening to the experiences of others, patients can gain emotional comfort and psychological support, enhancing their confidence and motivation to fight against diseases.^[23,24]^

This study is a retrospective study with a limited sample size. In the future, a large-sample multicenter randomized controlled trial will be conducted to further validate the effectiveness of narrative nursing interventions. It is meaningful to explore the impact of narrative nursing interventions on patients undergoing different types of surgeries and to develop more standardized and regulated narrative nursing intervention programmer.

## 5. Conclusion

In summary, narrative nursing has significant effects on improving postoperative anxiety, depression, pain levels, and QOL in patients with laparoscopic uterine fibroids. During the nursing period, personal experiences and patient stories can be used as the main nursing resources. The narrative process can strengthen communication between patients and nursing staff, promote patient understanding and recognition of the status of the disease, and is worthy of clinical promotion.

## Author contributions

**Conceptualization:** Xiaofeng Long, Cai Tang, Liqin Zhu.

**Data curation:** Yi Zhou.

**Formal analysis:** Yi Zhou, Liqi Zhang.

**Investigation:** Xiaofeng Long, Cai Tang, Huanyu Liang.

**Methodology:** Huanyu Liang.

**Validation:** Liqi Zhang, Liqin Zhu.

**Writing – original draft:** Xiaofeng Long, Cai Tang, Liqin Zhu.

## Supplementary Material


