# Supplementary material for: The impact of narrative nursing intervention on nursing outcomes of patients undergoing laparoscopic uterine fibroid surgery: A retrospective study
Source: Medicine (Baltimore). 2025 Sep 12;104(37):e44505. doi: 10.1097/MD.0000000000044505 (PMC12440445; doi:10.1097/MD.0000000000044505)
Supplement: Supplementary file 1 [file medi-104-e44505-s001.docx]

**Walking with You in the Light: A Seven Day Healing Journey for a Uterine Fibroid Patient**

**On admission**

On a cold winter morning in 2024, 39 year old Ms. Yang walked into the gynecology ward alone. She was wearing a slightly thin cotton coat, tightly holding a stack of inspection reports in her hand, with a mixture of anxiety and anticipation in her eyes. Six months ago, she accidentally felt a hard lump in her lower abdomen while lying flat. Now she has been diagnosed with multiple uterine fibroids and needs surgical treatment.

-“The 21st bed has arrived, this is your bed. "Nurse Zhou warmly approached, but found that the middle-aged woman did not respond immediately. Instead, she looked around, her gaze lingered on the ward facilities for a long time, and finally landed on the expense list on the bedside table.

-“Nurse, how much does this surgery cost? "Ms. Yang's voice was very soft, her fingers unconsciously twisting the corners of her clothes. Nurse Zhou noticed her rough hands and washed white cuffs, and understood her concerns.

-“Auntie Yang, health is like everything, money can be earned slowly. "nurse Zhou pulled out a chair and sat next to her.

-"Our hospital has a medical insurance reimbursement policy, can I help you calculate it in detail”. Nurse Zhou explained with patience.

At the time of admission, it was established mutual trust between nurse and patient through communication, and got the information of the patient's income, work, education, and family situation, etc.

**The eve of surgery: anxiety caused by the dim moonlight**

At 10 pm in the hospital room, Ms. Yang rang the call bell for the third time. When the on duty nurse Zhou pushed open the door and entered, she saw her clutching the ultrasound report with anxiety on her face.

“Nurse, can a single port laparoscope cleanly remove such a large fibroid?” Her voice is like a taut string.

“I checked the information and found that multiple fibroids are prone to recurrence, and... " Ms. Yang continued to confide her concerns.

Nurse Zhou noticed that “the phone screen on the bedside table showed a search page for" HPV42 type and cervical cancer risk”.

Nurse Zhou sat down gently, her trembling hand covered with warm fingertips.

As a mother of two children, Nurse Zhou understood this fear too well including the questions about fertility, intimate relationships between couples, and cancer risk were now entwining around this young mother like vines.

-"When I gave birth to my second child, I also had my fibroids removed, "Nurse Zhou lifted her clothes and revealed the almost invisible scar on her belly button.

-" Look, with the current single hole technique, all the wounds are hidden here. "

Nurse Zhou described her experience and feelings during the surgery helped Ms. Yang understood the surgical process.

The moonlight shone through the screen and cast their silhouettes on the wall, as if fate and hope were playing a game.

**Surgical Morning Light: Between Professionalism and Warmth**

On the morning of November 12th, as the wheels of the transfer bed rolled over the corridor, Ms. Yang's tears finally burst.

-" My daughter is only five years old... "She choked up and grabbed Nurse Zhou's hand.

The surgical nurse was checking the medication when Nurse Zhou suddenly leaned over and whispered in her ear,

-"Remember you said your daughter likes Princess Elsa? Later, we will use blue disinfectant for you, just like Elsa's magic." This sudden fairy tale metaphor made Ms. Yang laugh and cry as she was pushed into the operating room.

**Postoperative Rebirth: Life Lessons in Pain**

When the ticking sound of the monitor became clear again, Ms. Yang’s first reaction was to touch her abdomen.

Nurse Zhou immediately understood and said,

-"The fibroid is 15 cm long, but the wound is only 2.5cm."

She turned on the flashlight on her phone and let the patient peek through the gauze to see the incision that would be hidden in the navel in the future. During the night ward round, Nurse Zhou brought two warm palace warming patches.

-"These are the secret weapons of traditional Chinese medicine," she said while demonstrating acupoint pressing, her fingertips circling around the Guanyuan acupoint, like a mother soothing a baby...

**The dawn under the cloud of HPV**

On the day when the pathological report was released, Nurse Zhou specially came with an illustrated HPV popular science manual.

-"Type 42 belongs to the low-risk category, just like the common cold virus. "She circled the normal results of TCT with a red pen.

-"Your immune system is fighting, we can 'reinforce' it together."

Then, the vitamin bottles and exercise schedules on the table gradually replaced the panic screenshots searched online.

**Embrace upon discharge**

On the morning of November 17th, while Ms. Yang was practicing her abdominal tightening movements in front of the mirror, Nurse Zhou pushed the door open while holding a sunflower.

-"The wound is healing well, but there are some things I need to tell you, sir. "She blinked mischievously and pulled out a pink card with the words

-"Postoperative Intimacy Guide. "

-" In three months, you will have a more relaxed intimate time than before.”

At the entrance of the ward, Ms. Yang suddenly turned around and bowed deeply. When Nurse Zhou helped her, she heard a tearful whisper:

-"Thank you for not turning me into the case number in the textbook."

The spring breeze blew away the petals, and those stories about fertility anxiety, cancer fear, and physical shame will eventually transform into witnesses of vitality in the interweaving of professional medical care and humanistic care.

After three months of follow-up, Ms. Yang's HPV test turned negative. She posted a family photo on her social media with the caption "Scar is a special medal given to me by life"

The most precious treatment in this medical care may have occurred in the late night handshake, fairy tale anesthesia metaphor, and the carefully prepared pink card that was shy to speak of. When nursing breaks through the realm of technology and truly reaches the core of patients' fears, the doctor-patient relationship becomes a life alliance that fights against fate together.
